# Model prediction of radioactivity levels in the environment and food around the world’s first AP 1000 nuclear power unit

**DOI:** 10.3389/fpubh.2024.1400680

**Published:** 2024-05-15

**Authors:** Peng Wang, Wengzhe Huang, Hua Zou, Xiaoming Lou, Hong Ren, Shunfei Yu, Jiadi Guo, Lei Zhou, Zhongjun Lai, Dongxia Zhang, Zhiqiang Xuan, Yiyao Cao

**Affiliations:** ^1^Zhejiang Provincial Center for Disease Control and Prevention, Hangzhou, Zhejiang, China; ^2^Yangming College, Ningbo University, Ningbo, Zhejiang, China

**Keywords:** radioactivity levels, AP1000, time-series analysis, ARIMA, food, environment

## Abstract

**Objectives:**

Model prediction of radioactivity levels around nuclear facilities is a useful tool for assessing human health risks and environmental impacts. We aim to develop a model for forecasting radioactivity levels in the environment and food around the world’s first AP 1000 nuclear power unit.

**Methods:**

In this work, we report a pilot study using time-series radioactivity monitoring data to establish Autoregressive Integrated Moving Average (ARIMA) models for predicting radioactivity levels. The models were screened by Bayesian Information Criterion (BIC), and the model accuracy was evaluated by mean absolute percentage error (MAPE).

**Results:**

The optimal models, ARIMA *(0, 0, 0) × (0, 1, 1)_4_*, and ARIMA *(4, 0, 1)* were used to predict activity concentrations of ^90^Sr in food and cumulative ambient dose (CAD), respectively. From the first quarter (Q1) to the fourth quarter (Q4) of 2023, the predicted values of ^90^Sr in food and CAD were 0.067–0.77 Bq/kg, and 0.055–0.133 mSv, respectively. The model prediction results were in good agreement with the observation values, with MAPEs of 21.4 and 22.4%, respectively. From Q1 to Q4 of 2024, the predicted values of ^90^Sr in food and CAD were 0.067–0.77 Bq/kg and 0.067–0.129 mSv, respectively, which were comparable to values reported elsewhere.

**Conclusion:**

The ARIMA models developed in this study showed good short-term predictability, and can be used for dynamic analysis and prediction of radioactivity levels in environment and food around Sanmen Nuclear Power Plant.

## 1 Introduction

As a clean energy with near zero emissions, nuclear power has been vigorously developed in China in recent years as one of the important measures to achieve carbon neutrality by 2060 (1). Currently, there are 55 nuclear reactors in operation, and 18 under construction in China (2). During the operation of nuclear power plants, radioactive debris or effluents are inevitably discharged into the environment through air and water. As the continuous expansion of nuclear energy, the levels of radioactivity in environment and food have become a major concern for residents around the nuclear power plant (3–5), especially after the Fukushima Daiichi Nuclear Power Plan accident in 2011.

Radioactive substances present in the environment could potentially induce radiation exposure to human via external radiation and internal radiation through absorption, inhalation, and ingestion. As per the findings of the United Nations Scientific Committee on the Effects of Atomic Radiation (UNSCEAR 2000), an estimated 8% of natural human radiation exposure can be attributed to the ingestion of water and food (6). In order to safeguard the well-being of inhabitants, the World Health Organization (WHO) has established thresholds for gross alpha (0.5 Bq/L) and gross beta (1.0 Bq/L) in drinking water as a means of ensuring radiation safety (7). ^90^Sr is a high-yield byproduct of nuclear fission (8, 9), possessing a half-life of 28.8 years. Its primary route of entry into human body is through the food chain, where it can accumulate in teeth, bones, and muscle tissues. Given its relatively long half-life and high radiotoxicity, ^90^Sr is recognized as a crucial artificial radionuclide for evaluating radiation risks to both the environment and human health (10, 11).

In view of the radiation exposure risks of and high level of public concern, prediction of radioactivity levels in the environment and food around the nuclear power plant are crucial to ensure radiation safety for the public and the environment.

Time series analysis is widely recognized as a valuable predictive model, and the Autoregressive Integrated Moving Average model (ARIMA) being one of the most important models. The ARIMA model, a hybrid of autoregressive and moving average models, was initially proposed in 1976 by Box and Jenkins (12). So far, it has been widely used in economic management, meteorological prediction, environmental prediction, and disease prediction (13–16). In addition, ARIMA model also has been applied in the field of radioactive monitoring and evaluation. In four districts of Istanbul, the model was used to predict concentrations of ^226^Ra, ^232^Th, and ^40^K (17). After Fukushima Daiichi Nuclear Power Plan accident, Hemn Salh et al. reported the use of ARIMA models for prediction of air radiation dose rates around (18). Some scholars have already used it to predict radon concentrations and thus to predict earthquakes (19, 20).

Sanmen Nuclear Power Plant (SNPP), located in Sanmen, Zhejiang, China, adopts the world’s most advanced third-generation pressurized water reactor (AP1000) technology, which is one of the achievements of China’s efforts to develop nuclear power. With the operation of SNPP, its impacts on the environment and residents’ health have become a growing concern. To assess the impacts, we have continuously monitored radioactivity levels in the environment and food around SNPP since 2011. In this study, we analyzed the historical monitoring data and used ARIMA models, which is a time-series analysis technology, to fit and predict radioactive levels around SNPP for the first time. The predicted data can provide a basis for environmental impact assessment and human health risk assessment around SNPP.

## 2 Data and methods

### 2.1 The study region

**Figure 1:**
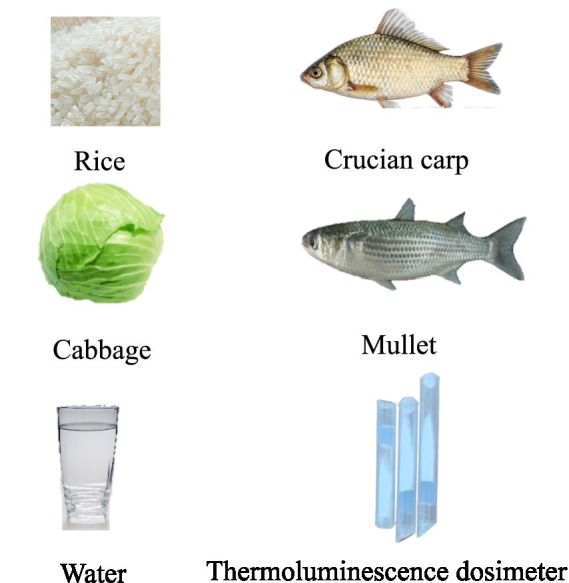
Samples collected in this study.

**Figure 2:**
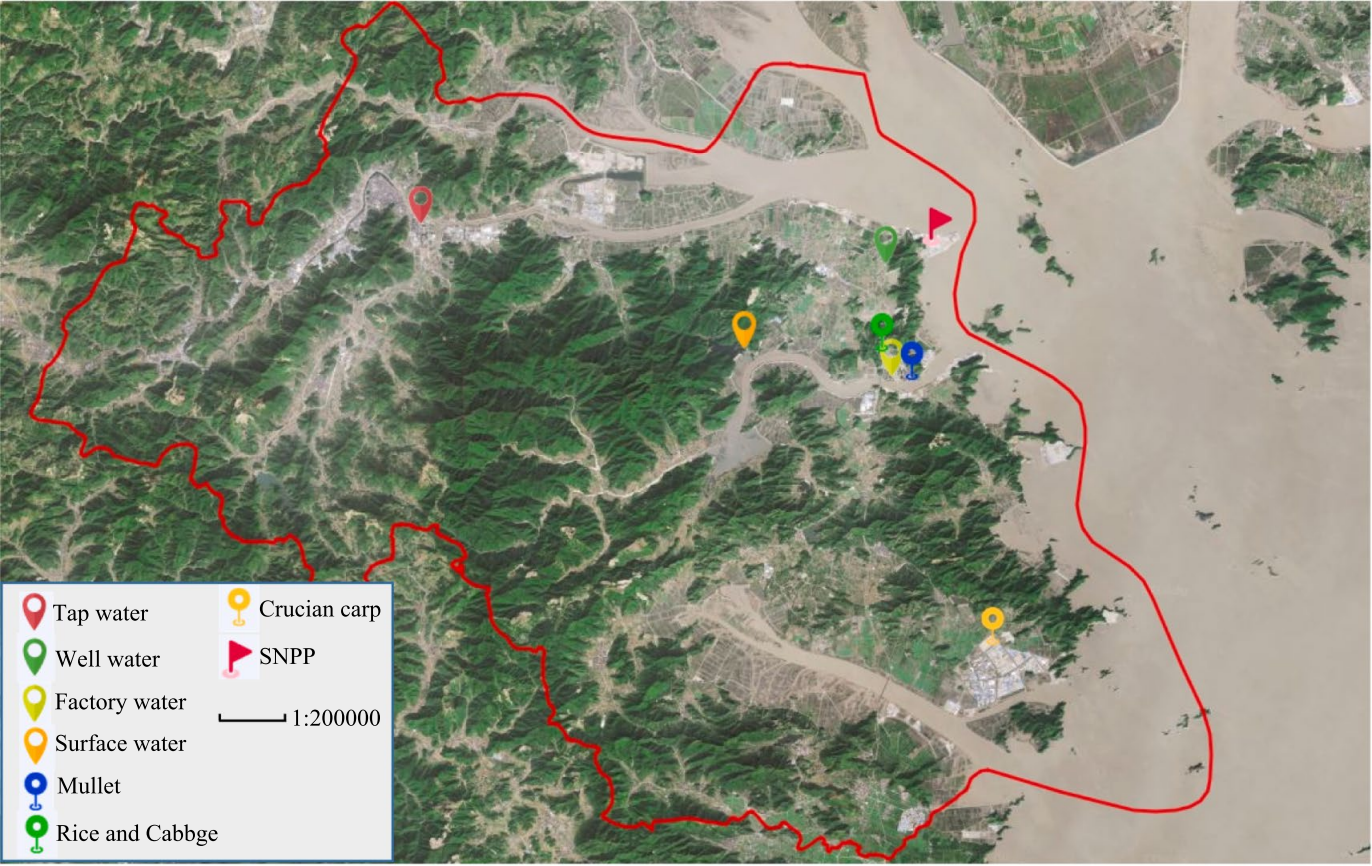
Monitoring stations of water and food around SNPP. The map was produced by software of Lantu Draw (URL link: https://www.ldmap.net/).

**Figure 3:**
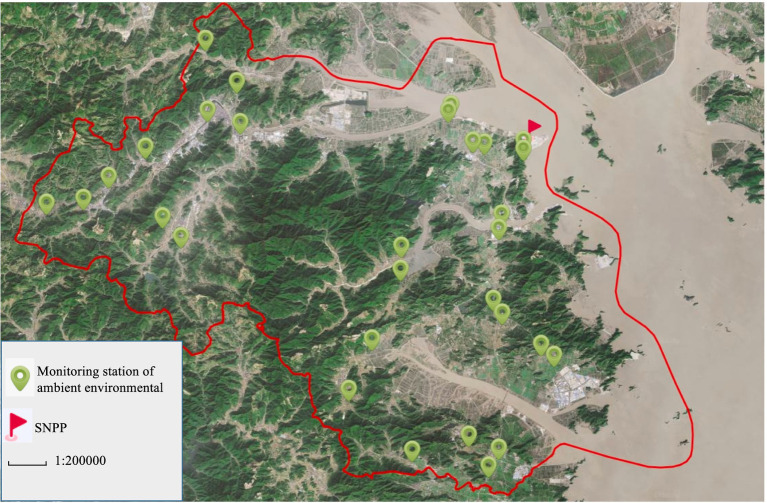
Monitoring stations for ambient radiation exposure of ambient environmental around SNPP. The map was produced by software of Lantu Draw (URL link: https://www.ldmap.net/).

All monitoring stations were located in Sanmen County. There were four monitoring stations for gross *α* and gross *β* in water representing surface water, factory water, tap water, and well water, respectively. Water samples were collected quarterly. There were three monitoring stations for ^90^Sr in collected quarterly food including mullet, crucian carp, cabbage, and rice. There were 30 monitoring stations for ambient radiation, which were monitored quarterly. The images of the collected samples are shown in Figure 1. All data were obtained from Zhejiang Provincial Center for Disease Control and Prevention and Taizhou City Center for Disease Control and Prevention. In this study, all these monitoring data were averaged across the corresponding monitoring stations as shown in Figures 2, 3. The dataset of gross *α* in water was excluded because the activity concentrations were mostly below the detection limits.

### 2.2 Sample preparation and analysis

#### 2.2.1 Gross *α* and *β* in water

**Figure 4:**
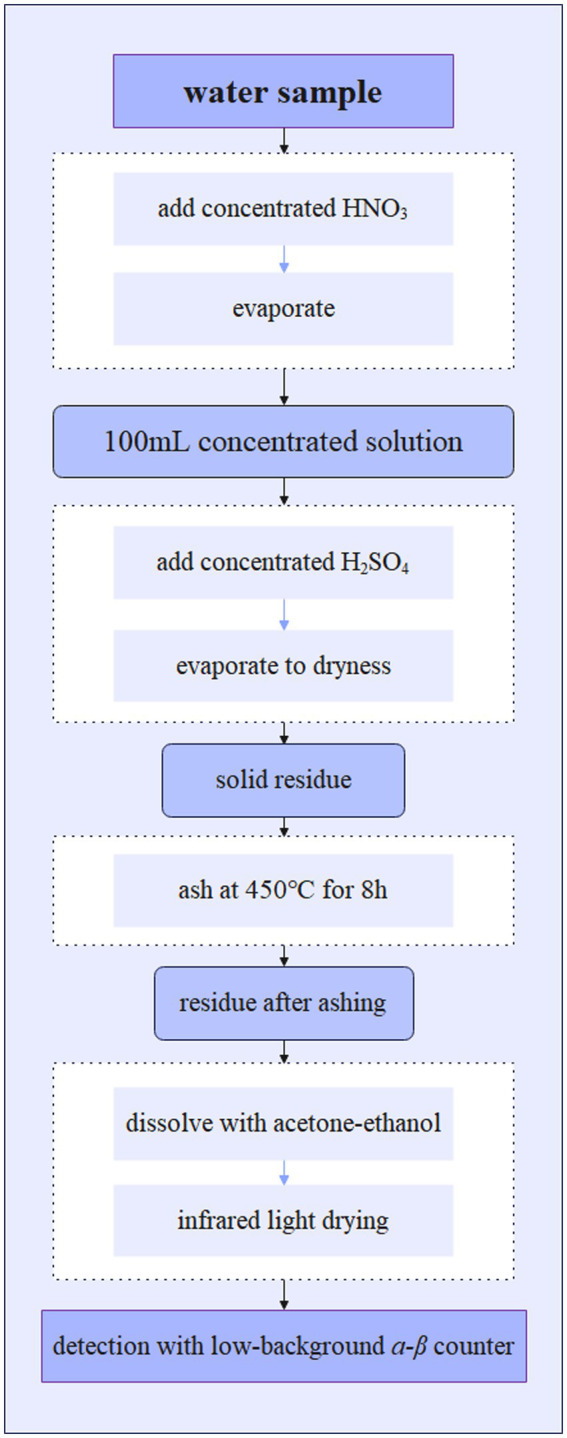
Procedure for determination of gross *α* and *β* in water.

Figure 4 illustrates the schematic flow for determination of gross *α* and *β* in water, according to the Chinese national standard (21).

#### 2.2.2 ^90^Sr in food

**Figure 5:**
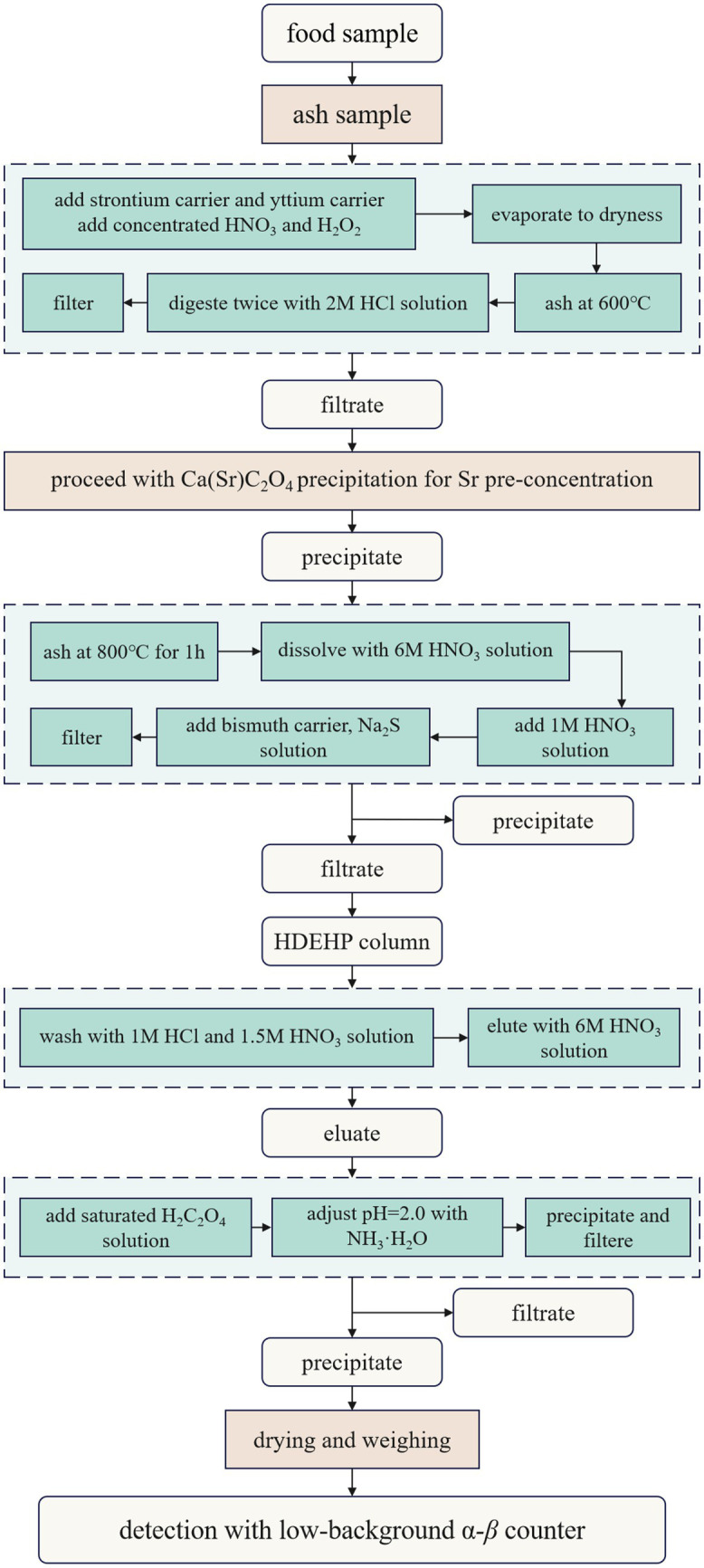
Procedure for determination of ^90^Sr determination in food.

Figure 5 illustrates the schematic flow for determination of ^90^Sr in food, according to the Chinese national standard (22).

#### 2.2.3 Ambient radiation

The cumulative ambient dose (CAD) was monitored by thermoluminescent dosimeter (TLD) (23). The LiF (Mg, Cu, and P) powder was placed into a hermetically sealed container in order to fabricate TLD. Each monitoring point was equipped with two TLDs installed at a height of 2 m. The TLDs were measured by TLD reader.

### 2.3 Data

The model training dataset (Supplementary Tables S1–S3) included ^90^Sr activity concentrations in food from the second quarter (Q2) of 2011 to the fourth quarter (Q4) of 2022, gross *β* activity concentrations in water from the first quarter (Q1) of 2016 to Q4 of 2022 and CAD from Q1 of 2011 to Q4 of 2022. The monitoring data from Q1 to Q4 in 2023 were used as a test dataset to assess the predictability of the models using the mean absolute percentage error (MAPE) between the forecasted and observed values. Finally, the best model was applied to forecast the radioactivity levels from Q1 to Q4 of 2024.

### 2.4 Methods

#### 2.4.1 Auto-regressive integrated moving average [ARIMA (*p*,*d*,*q*)]

An ARIMA model is defined by three parameters: *p, d,* and *q*, where *p* is the order of Auto-Regressive (AR) term, *d* is the order of differencing required to make the time-series stationary, and *q* is the order of Moving Average (MA) term.

AR (*p*) usually explains the present value *X_t_*, unidirectionally it terms of its previous values *X_t − 1_,X_t − 2_,, X_t − p_*, and the current residuals *ε_t_*. It can be expressed as Equation (1):


(1) $$X_{t}=\phi_{1}X_{t-1}+\phi_{2}X_{t-2}+\cdots +\phi_{p}X_{t-p}+\varepsilon_{t}$$

The model illustrates a linear association between the current observed value of the sequence at time *t* and the past observed values at the preceding *p* time points. This type of regression was known as autoregression because it is based on its own historical data. The regression with the observed values from the previous *p* time points is referred to as *p*-order autoregression. *φ_i_* (i = 1, 2…*p*) is its partial regression coefficient.

MA (*q*) refers to the current value of the time series *X_t_* in terms of its current and previous residuals *ε_t − 1_*, *ε_t − 2_*,…, *ε_t − q_*. It can be expressed as Equation (2):


(2) $$X_{t}=\varepsilon_{t}-\theta_{1}\varepsilon_{t-1}-\theta_{2}\varepsilon_{t-2}-\cdots -\theta_{q}\varepsilon_{t-q}$$

The model indicates that the value of the sequence at time *t* is independent of the past observed values at the preceding *q* time points, however, it exhibits a linear relationship with the preceding *q* stochastic disturbances. Therefore, the model is referred to as the *q*-order moving average model. The *ε_t_* is indicative of a stochastic disturbance sequence, also referred to as a white noise sequence, that is characterized by independence and adherence to a normal distribution. *θ_i_* (i = 1, 2…*q*) is its partial regression coefficient.

The ARIMA model is the combination of AR model and MA model algorithms. I in the ARIMA (*p,d,q*) refers to Integrated. When time-series is stationary, the ARIMA (*p,d,q*) model is ARMA *(p,q)*, can be expressed as Equation (3):


(3) Xt=ϕ1Xt−1+ϕ2Xt−2+⋯+ϕpXt−p+εt−θ1εt−1−θ2εt−2−⋯−θqεt−q

Transform the Equation (3) to Equation (4).


(4) Xt−ϕ1Xt−1−ϕ2Xt−2−⋯−ϕpXt−p=εt−θ1εt−1−θ2εt−2−⋯−θqεt−q

In order to facilitate ease of expression, a backshift operator is employed, which is akin to a time pointer. The multiplication of the present sequence value by a backshift operator is tantamount to shifting the temporal position of said sequence value one moment into the past. If denoted as *B*, the backshift operator yields Equation (5) and (6):


(5) $$B^{m}X_{t}=X_{t-m}$$

(6) $$B^{m}\varepsilon_{t}=\varepsilon_{t-m}$$

Upon utilization of the backshift operator, Equation (4) is transformed to Equation (7).


(7) $$X_{t}=\frac{\theta \left(B\right)}{\phi \left(B\right)}\varepsilon_{t}$$

Where *φ(B)* is non-seasonal autoregressive polynomial, *θ(B)* is non-seasonal movingaverage polynomial, which are expressed as Equation (8) and (9):


(8) $$\varphi \left(B\right)=1-\varphi_{1}B-\varphi_{2}B^{2}-..-\varphi_{p}B^{p}$$

(9) $$\theta \left(B\right)=1-\theta_{1}B-\theta_{2}B^{2}-..-\theta_{q}B^{q}$$

If the time-series is non-stationary, which should be converted to stationary by differencing, the model used is ARIMA (*p,d,q*). Assume Δ*X_t_* is the sequence which obtained after first-order differencing of *X_t_*, which is expressed as Equation (10):


(10) $$\Delta X_{t}=X_{t}-X_{t-1}$$

Therefore, the equation of ARIMA (*p,d,q*) as follows Equation (11), which transforms from Equation (7).


(11) $$\Delta^{d}X_{t}=\frac{\theta \left(B\right)}{\phi \left(B\right)}\varepsilon_{t}$$

#### 2.4.2 Multiple seasonal auto-regressive integrated moving average [MSARIMA *(p, d, q) (P, D, Q)*]

The Seasonal-ARIMA [SARIMA*(p, d, q)(P, D, Q)*] model incorporates a non-seasonal ARIMA*(p, d, q)* model along with supplementary seasonal terms *(P, D, Q)_s_*, which accounts for the seasonality inherent in the time-series data over S time steps, corresponding to a singular seasonal period. *P* is the order of seasonal autoregressive term, *D* is the order of seasonal differencing, *Q* is the order of seasonal moving average term and *S* is the length of the seasonal cycle. The complete expression of the SARIMA model can be written as Equation (12):


(12) $$\Delta^{d}\Delta_{S}^{D}X_{t}=\frac{\theta \left(B\right)V\left(B^{s}\right)}{\phi \left(B\right)U\left(B^{s}\right)}\varepsilon_{t}$$

Where *ε_t_* was white noise; *U(B^S^)* is seasonal autoregressive polynomial, and *V(B^S^)* is seasonal moving average polynomial, which are expressed as Equation (13) and (14):


(13) $$U\left(B^{S}\right)=1-u_{1}B^{S}-u_{2}B^{2S}-..-u_{P}B^{PS}$$

(14) $$V\left(B^{S}\right)=1-v_{1}B^{S}-v_{2}B^{2S}-..-v_{Q}B^{QS}$$

When *P* = *Q* = *D* = 0, it means that there is no seasonal in the model. In this case, the model reduces to the standard ARIMA model.

## 3 Results and discussion

### 3.1 Model establishment

**Figure 6:**
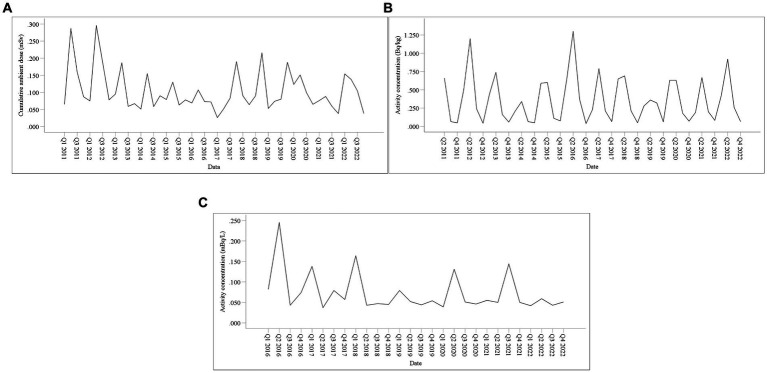
Time-series diagram of monitoring data. **(A)** CAD; **(B)** ^90^Sr activity concentrations in food; **(C)** Gross *β* activity concentrations in water.

**Figure 7:**
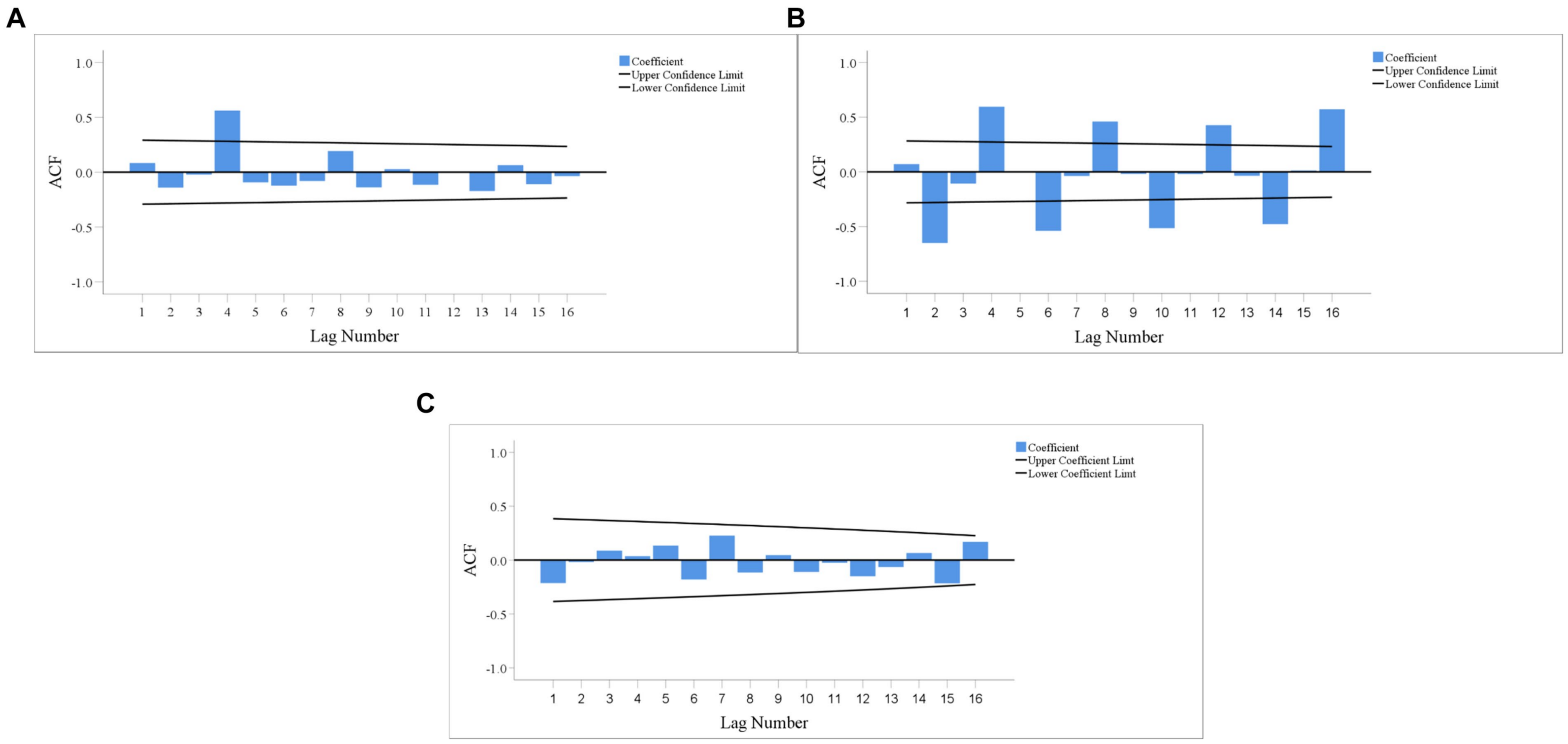
ACF plot of original sequence. **(A)** CAD; **(B)** ^90^Sr activity concentrations in food; **(C)** Gross *β* activity concentrations in water.

Figures 4, 5 show the time-series diagrams and autocorrelation function (ACF) plots of the original sequences of the CAD, ^90^Sr activity concentrations in food, and gross *β* activity concentrations in water. The time-series diagram for the CAD (Figure 6A) shows fluctuations within a certain range and most ACFs (Figure 7A) fall into the confidence interval and tend to zero rapidly. This indicates that the sequence is stable and does not need differencing. In contrast, both the time-series diagrams (Figure 6B) and ACF plots (Figure 7B) for the ^90^Sr activity concentrations in food exhibit clear periodic changes, indicating that the sequences require seasonal differencing. For gross *β* activity concentrations in water, the time series diagram (Figure 6C) appears relatively stable without any obvious trend, but all ACFs (Figure 7C) fall within the confidence interval. Therefore, it was determined to be white noise, and further analysis was halted.

**Figure 8:**
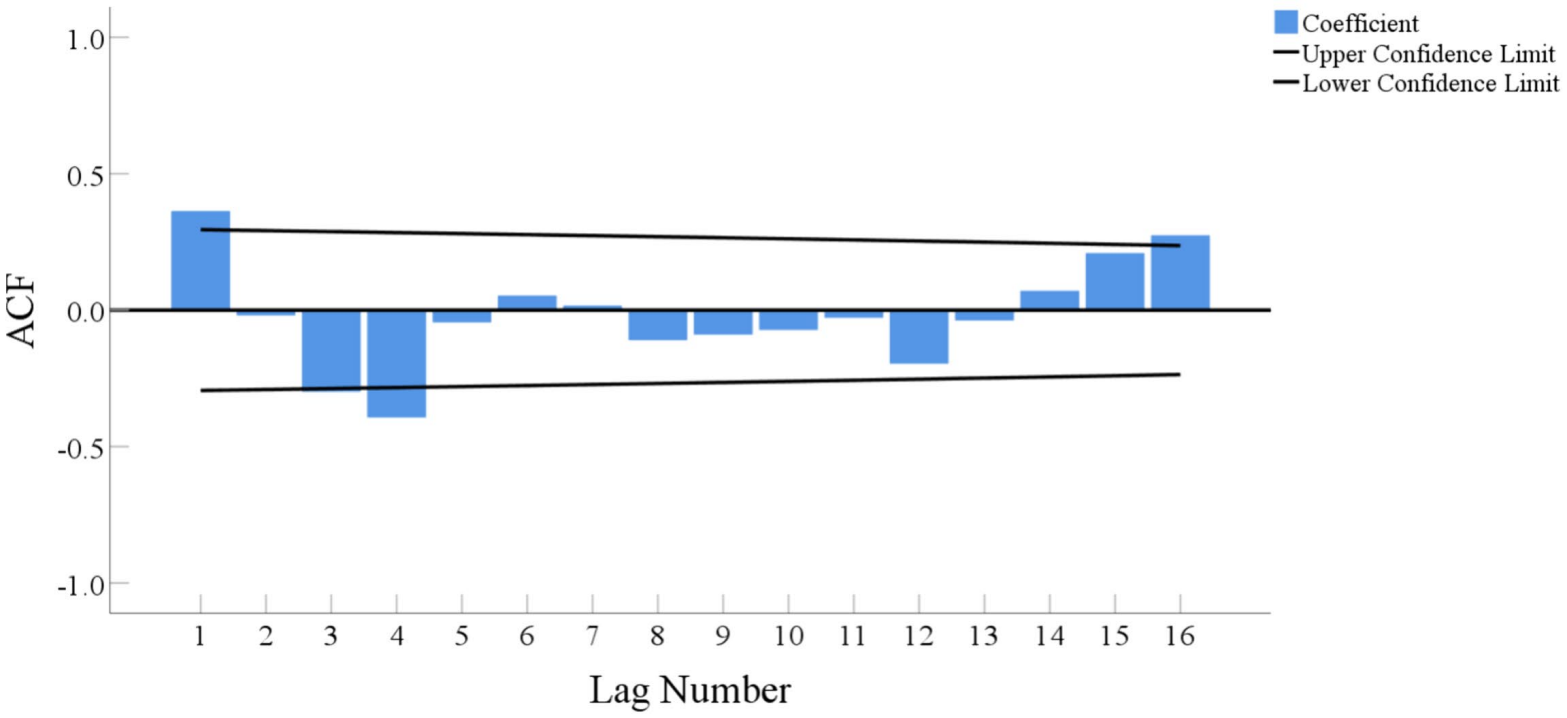
ACF plot of the sequence of ^90^Sr activity concentrations in food after performing first-order seasonal differencing.

After performing first-order seasonal differencing on the original time-series of ^90^Sr activity concentrations in food, the ACF plot of the resulting time-series is shown in Figure 8. The majority of ACFs fall within the confidence interval and quickly reach zero, indicating that the new sequence is stable and does not exhibit any significant seasonal fluctuations.

The determination of *p, q, P* and *Q* is a crucial aspect for establishing the ARIMA model. Some researchers suggested that the most effective method to determine these values involves analyzing ACF and partial autocorrelation function (PACF) of time-series after differencing and seasonal differencing. The lag number of the peak entering the confidence level in ACF and PACF plots is used to determine the values of *p, q, P* and *Q* (24–28). However, some scientists criticized this estimation method for not being sufficient every time (29). Besides the ACF and PACF plots, there are other methods that can be used to determine the optimal ARIMA model, such as the Bayesian Information Criterion (BIC). The model with the lowest BIC value is considered as the best fit.

In this study, we tested various parameter values for *p, q, P* and *Q* (with a maximum value of 4) in ascending order, and selected the models with the lowest BIC values. After model identification and parameter estimation, we selected the models that met all standards, as shown in Table 1.

**Table 1:** Optimal models.

Name	Optimal model	BIC	Ljung-Box Q-test
			*p* values
^90^Sr in food	ARIMA(0, 0, 0) × (0, 1, 1)_4_	−3.024	0.388
CAD	ARIMA(4, 0, 1)	−5.532	0.927

Another key point of model construction is the diagnosis of residual sequence and model parameters. If the residual sequence of the model follows a normal distribution and appears random like white noise, it suggests that the model has extracted most of the information from the original sequence (30). The residual sequences that are considered as white noise could be verified by Ljung-Box Q-test (31, 32) with *p* value greater than 0.05. In addition, the parameters of the model should also be significantly different from zero (*p* < 0.05) to ensure the validity of the model. The models shown in Table 1 all passed both residual sequences Ljung-box Q-test and model parameters diagnosis, which were eventually selected as the optimal models.

### 3.2 Model fitting and prediction

**Figure 9:**
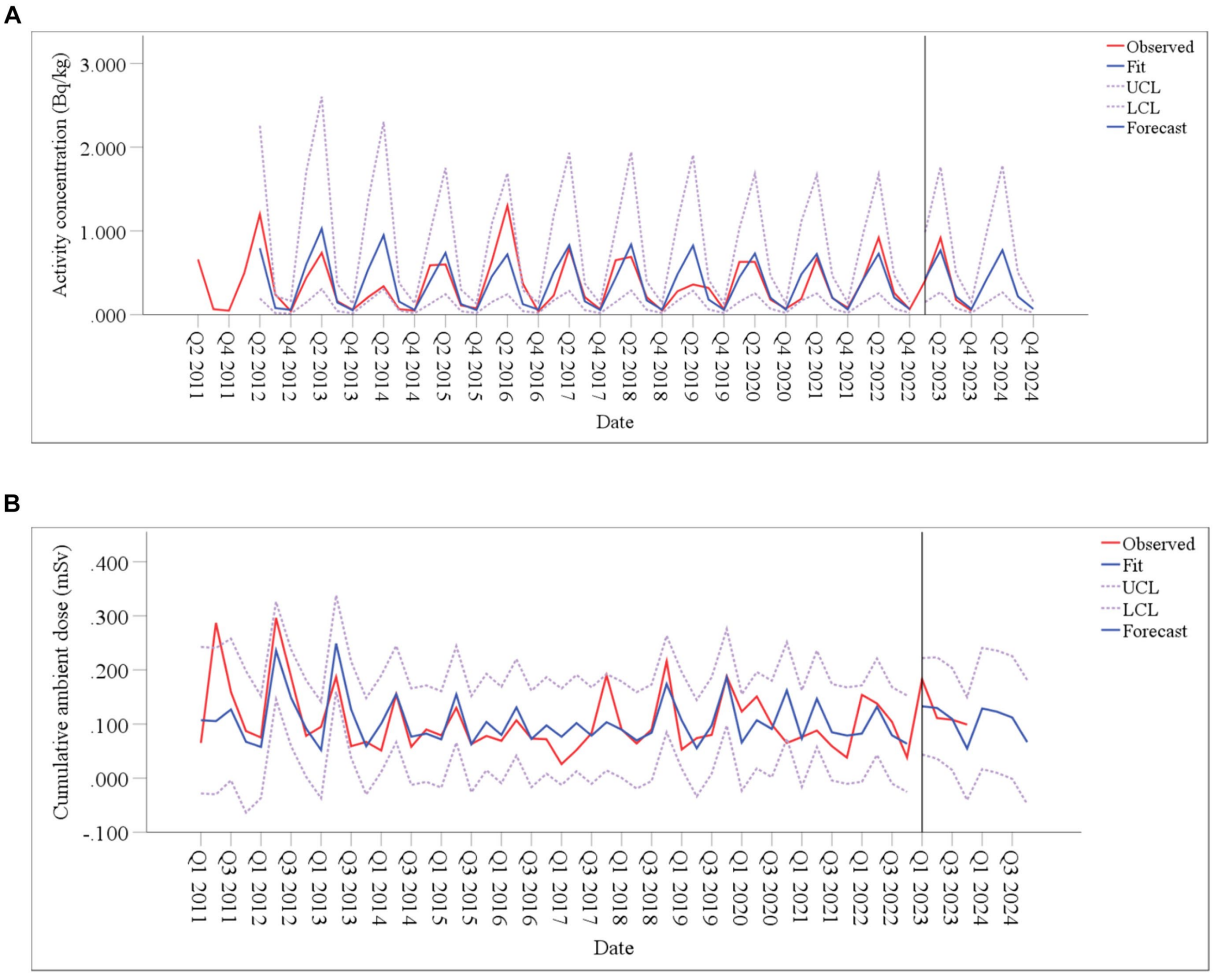
Time-series diagram for the model fit and forecast. **(A)** ^90^Sr activity concentrations in food; **(B)** CAD.

Figure 9 shows the model forecasting results covering the training, testing and prediction periods. Each diagram consists of the observed, fitted and forecasted data, as well as the upper and lower confidence limits. The fitted values in the diagram follow the same trend as the observed values and are within the confidence interval, indicating that the fitting effect of the model is good. To further the accuracy of the model, MAPEs were calculated between the observed and the forecast values for ^90^Sr activity concentrations in food, CAD from Q1 to Q4 in 2023, which were 21.4 and 22.4%, respectively (Table 2). These low error values indicate that the model has a high level of accuracy in predicting the future values of these variables.

**Table 2:** Observed and predicted radioactivity levels around SNPP from Q1 to Q4 in 2023.

Quarter	Observed value		Forecast value		95% confidence interval		Absolute percentage error	
	^90^Sr activity concentration in food (Bg/kg)	CAD (mSv)	^90^Sr activity concentration in food (Bg/kg)	CAD (mSv)	^90^Sr activity concentration in food (Bg/kg)	CDA (mSv)	^90^Sr activity concentration in food (%)	CAD (%)
1	0.40	0.183	0.43	0.133	0.150–0.984	0.044–0.222	7.5	27.3
2	0.92	0.111	0.77	0.130	0.270–1.764	0.036–0.223	16.3	17.1
3	0.18	0.108	0.22	0.109	0.076–0.497	0.015–0.204	22.2	0.9
4	0.048	0.099	0.067	0.055	0.024–0.154	−0.040-0.150	39.6	44.4
Mean absolute percentage error (MAPE)							21.4	22.4

In this study, the established optimal models were applied to forecast the radioactivity levels around SNPP in 2024. As shown in Table 3, the activity concentrations of ^90^Sr in food from Q1 to Q4 were predicted to be 0.067–0.77 Bq/kg, and the CAD to be 0.067–0.129 mSv. The forecasted values of ^90^Sr in food are lower than the concentration limit recommended by the Chinese standard (33) and comparable to the activity concentrations of ^90^Sr in food from other regions of the world (Table 4) (34–40). The forecasted values of CAD are comparable to the level of Qinshan Nuclear Power Plant (0.073–0.093 mSv) (41).

**Table 3:** Forecast values of radioactivity levels around SNPP from Q1 to Q4 in 2024.

Quarter	Forecast value	
	^90^Sr activity concentration in food (Bq/kg)	CAD (mSv)
1	0.43	0.129
2	0.77	0.123
3	0.22	0.112
4	0.067	0.067

**Table 4:** Comparison of activity concentrations of ^90^Sr in foods from different regions of the world.

Location	Sample type(sample time)	Activity concentration (Bq/kg)
Vicinity of Ningde Nuclear Power Plant, China (34)	Rice, Vegetables, Marine fish, Freshwater fish (2013–2017)	<0.017–0.677
Qinshan Nuclera Power Plant, China (35)	Rice, Salsola, Mullet, Crucian carp (2012–2019)	0.04–1.3
Tianwan Nuclear Power Plant, China (36)	Rice, Chinese cabbage, wheat (March 2000 to April 2002)	0.024–0.23
Cuban (37)	Vegetables, Fish (/)	0.0034–0.28
Austrian (38)	Cereals, Cabbage, Freshwater fish (1997)	0.09–0.12
Mayak Industrial Association (39)	Wheat, Cabbage, Carrots (2008–2010)	0.029–0.24
Niigata, Japan (40)	Undaria pinnatifida (1999–2007)	<0.016–0.036

Based on radioactivity monitoring data around SNPP, ARIMA models were established to fit and predict the short-term changes. However, there were instance where the forecast values and actual values of individual quarters differ greatly, such as ^90^Sr in food in Q1 and Q2 of 2019, CAD in Q4 of 2017 and 2020, and Q2 of 2021 (Figure 7). These differences may be related to large fluctuations of external conditions, such as climate (42), which were accounted by the ARIMA model. While ARIMA models focus on the role of time factors in fitting and forecasting, they do not analyze and discuss the relationship between the prediction object and the influencing factors (43).

When the prediction time becomes longer, these external influencing factors bring greater changes. Therefore, despite ARIMA models can maintain high accuracy when using historical data for short- and medium-term prediction, their accuracy may decrease when predicting further into the future. To improve the models accuracy in predicting radioactivity levels around SNPP for a longer period, continuous radioactivity monitoring data collection should be carried out to adjust and refine the model over time (44). This will ensure that the models reflect the variability and trends of radioactivity levels, leading to the best possible predictions.

In this study, the monitoring data from 2011 to 2022 as the training dataset. In June 2018, after the operation of SNPP, significant changes have taken place in external conditions. In our previous research, we found that the contributions of radioactive substances released to the environment after SNPP operation was negligible (45, 46), we think this change does not affect the establishment of the model. In addition, if a nuclear accident or other unknown external input occurs, the model cannot predict. When the observed data are significantly higher than predicted, excluding external input, it indicates that SNPP significantly releases radioactive substances.

The ARIMA model has been successfully applied to SNPP, but its applicability to other nuclear power plants with different technologies, operating practices or environmental backgrounds, such as Qinshan Nuclear Power Plant, will depend on the regularity and randomness of the time series of their historical radioactivity data. If the time series has a certain regularity and norandom, the ARIMA model has good potential to be applied.

## 4 Conclusion

This study attempts to develop ARIMA models for medium and short-term prediction of radioactive levels in the environment and food around SNPP, utilizing historical time series data spanning from 2011 to 2023. The data from 2011 to 2022 were used as the training set, while the data from 2023 served as the testing set. The time series of gross *β* in water was a white noise series, which has no value in establishing a model. In contrast, the time series of ^90^Sr in food and CAD were stationary, non random, and have short-term correlation, making them suitable for establishing ARIMA models. In this study, our established models showed good consistency between the fitted values and observed values, coupled with the relatively small MAPEs, suggesting satisfactory fitting effect and accuracy in the ARIMA models.

The CAD and ^90^Sr activity concentrations in food in 2024 were predicted using the established model. The forecasted values of ^90^Sr in food are lower than the recommended threshold by the Chinese standard, and comparable to the active concentrations of ^90^Sr in food from other regions of the world. The forecasted values of CAD are comparable to the level of Qinshan Nuclear Power Plant.

However, we acknowledge the limitation of the ARIMA models, such as some external factors, e.g., climate, were not taken into account. This limitation might be compensated by continuous feeding of monitoring data thus to adjust and improve the models performance over time. In short, ARIMA models can be used as an additional tool for environmental radioactivity monitoring and human health risk assessment around SNPP.

## Data availability statement

The original contributions presented in the study are included in the article/Supplementary material; further inquiries can be directed to the corresponding author.

## Author contributions

PW: Conceptualization, Methodology, Project administration, Software, Writing – original draft, Writing – review & editing. WH: Data curation, Investigation, Software, Writing – original draft. HZ: Supervision, Writing – review & editing. XL: Supervision, Writing – review & editing. HR: Data curation, Investigation, Methodology, Writing – original draft. SY: Data curation, Investigation, Writing – original draft. JG: Data curation, Investigation, Writing – original draft. LZ: Data curation, Investigation, Writing – original draft. ZL: Data curation, Writing – original draft. DZ: Data curation, Writing – original draft. ZX: Writing – original draft, Formal analysis. YC: Supervision, Writing – review & editing.
