# Supplementary material for: Model prediction of radioactivity levels in the environment and food around the world’s first AP 1000 nuclear power unit
Source: Front Public Health. 2024 May 15;12:1400680. doi: 10.3389/fpubh.2024.1400680 (PMC11133631; doi:10.3389/fpubh.2024.1400680)
Supplement: Supplementary file 1 [file Data_Sheet_1.DOC]

**Supplementary Material** for

**Model Prediction of Radioactivity Levels in the Environment and Food around the World’s First AP 1000 Nuclear Power Unit**

**Table S1** Cumulative Ambient Dose around SNPP form 2011 to 2023

| Year | | Cumulative Ambient Dose (mSv) | | | |
| --- | --- | --- | --- | --- | --- |
|  |  | 1st Quarter | 2nd Quarter | 3rd Quarter | 4th Quarter |
| 2011 | value | 0.065 | 0.287 | 0.159 | 0.087 |
|  | sd | 0.038 | 0.135 | 0.109 | 0.056 |
| 2012 | value | 0.075 | 0.296 | 0.187 | 0.078 |
|  | sd | 0.089 | 0.145 | 0.121 | 0.042 |
| 2013 | value | 0.095 | 0.187 | 0.059 | 0.067 |
|  | sd | 0.036 | 0.048 | 0.012 | 0.021 |
| 2014 | value | 0.051 | 0.155 | 0.058 | 0.090 |
|  | sd | 0.039 | 0.085 | 0.021 | 0.019 |
| 2015 | value | 0.079 | 0.130 | 0.063 | 0.078 |
|  | sd | 0.032 | 0.088 | 0.049 | 0.035 |
| 2016 | value | 0.069 | 0.107 | 0.073 | 0.072 |
|  | sd | 0.019 | 0.029 | 0.025 | 0.038 |
| 2017 | value | 0.026 | 0.052 | 0.083 | 0.190 |
|  | sd | 0.0095 | 0.018 | 0.059 | 0.108 |
| 2018 | value | 0.090 | 0.064 | 0.090 | 0.216 |
|  | sd | 0.089 | 0.068 | 0.074 | 0.109 |
| 2019 | value | 0.053 | 0.074 | 0.080 | 0.188 |
|  | sd | 0.045 | 0.065 | 0.042 | 0.103 |
| 2020 | value | 0.123 | 0.151 | 0.099 | 0.065 |
|  | sd | 0.089 | 0.102 | 0.078 | 0.060 |
| 2021 | value | 0.076 | 0.088 | 0.059 | 0.038 |
|  | sd | 0.092 | 0.054 | 0.045 | 0.026 |
| 2022 | value | 0.154 | 0.138 | 0.104 | 0.038 |
|  | sd | 0.078 | 0.089 | 0.079 | 0.035 |
| 2023 | value | 0.183 | 0.111 | 0.108 | 0.099 |
|  | sd | 0.085 | 0.074 | 0.067 | 0.045 |

**Table S2 ^90^Sr activity concentrationsin in food samples around SNPP form 2011 to 2023**

| Year | | 1st Quarter | 2nd Quarter | 3rd Quarter | 4th Quarter |
| --- | --- | --- | --- | --- | --- |
| [Mullet](../../../../C:/Program%20Files%20(x86)/Youdao/Dict/9.0.4.0/resultui/html/index.html#/javascript:;)  (Bq/kg) | [Crucian carp](../../../../C:/Program%20Files%20(x86)/Youdao/Dict/9.0.4.0/resultui/html/index.html#/javascript:;)  (Bq/kg) | Cabbage  (Bq/kg) | Rice  (Bq/kg) |
| 2011 | value | - | 0.66 | 0.065 | 0.048 |
| sd | - | 0.055 | 0.0082 | 0.0060 |
| 2012 | value | 0.50 | 1.2 | 0.24 | 0.042 |
| sd | 0.033 | 0.23 | 0.014 | 0.0047 |
| 2013 | value | 0.44 | 0.74 | 0.16 | 0.058 |
| sd | 0.043 | 0.139 | 0.010 | 0.0044 |
| 2014 | value | 0.21 | 0.34 | 0.065 | 0.049 |
| sd | 0.031 | 0.039 | 0.0051 | 0.0048 |
| 2015 | value | 0.59 | 0.60 | 0.11 | 0.075 |
| sd | 0.041 | 0.068 | 0.010 | 0.0042 |
| 2016 | value | 0.64 | 1.3 | 0.37 | 0.037 |
| sd | 0.049 | 0.17 | 0.017 | 0.0040 |
| 2017 | value | 0.23 | 0.79 | 0.21 | 0.064 |
| sd | 0.044 | 0.063 | 0.017 | 0.0038 |
| 2018 | value | 0.65 | 0.69 | 0.21 | 0.049 |
| sd | 0.055 | 0.055 | 0.011 | 0.0061 |
| 2019 | value | 0.28 | 0.36 | 0.32 | 0.061 |
| sd | 0.021 | 0.052 | 0.021 | 0.0052 |
| 2020 | value | 0.63 | 0.63 | 0.18 | 0.071 |
| sd | 0.046 | 0.059 | 0.015 | 0.0039 |
| 2021 | value | 0.19 | 0.67 | 0.20 | 0.082 |
| sd | 0.058 | 0.076 | 0.027 | 0.012 |
| 2022 | value | 0.42 | 0.92 | 0.26 | 0.065 |
| sd | 0.078 | 0.14 | 0.039 | 0.011 |
| 2023 | value | 0.40 | 0.92 | 0.18 | 0.048 |
| sd | 0.12 | 0.095 | 0.011 | 0.004 |

**Table S3 Radioactivity of gross *α* and gross *β* in water around SNPP from 2016 to 2023**

| Year | | 1st Quarter | | 2nd Quarter | | 3rd Quarter | | 4th Quarter | |
| --- | --- | --- | --- | --- | --- | --- | --- | --- | --- |
|  |  | Gross *α*  (Bq/L) | Gross *β*  (Bq/L) | Gross *α*  (Bq/L) | Gross *β*  (Bq/L) | Gross *α*  (Bq/L) | Gross *β*  (Bq/L) | Gross *α*  (Bq/L) | Gross *β* (Bq/L) |
| 2016 | value | <0.016 | 0.082 | <0.016 | 0.245 | <0.016 | 0.043 | <0.016 | 0.074 |
|  | sd | - | 0.0052 | - | 0.204 | - | 0.023 | - | 0.018 |
| 2017 | value | <0.016 | 0.138 | <0.016 | 0.037 | <0.016 | 0.079 | <0.016 | 0.057 |
|  | sd | - | 0.143 | - | 0.013 | - | 0.040 | - | 0.0062 |
| 2018 | value | <0.016 | 0.164 | <0.016 | 0.043 | <0.016 | 0.047 | <0.016 | 0.045 |
|  | sd | - | 0.079 | - | 0.011 | - | 0.014 | - | 0.016 |
| 2019 | value | <0.016 | 0.079 | <0.016 | 0.052 | <0.016 | 0.044 | <0.016 | 0.054 |
|  | sd | - | 0.026 | - | 0.020 | - | 0.015 | - | 0.012 |
| 2020 | value | <0.016 | 0.039 | <0.016 | 0.131 | <0.016 | 0.051 | <0.016 | 0.046 |
|  | sd | - | 0.0065 | - | 0.059 | - | 0.011 | - | 0.030 |
| 2021 | value | <0.016 | 0.055 | <0.016 | 0.050 | <0.016 | 0.144 | <0.016 | 0.050 |
|  | sd | - | 0.012 | - | 0.0042 | - | 0.087 | - | 0.010 |
| 2022 | value | <0.016 | 0.042 | <0.016 | 0.059 | <0.016 | 0.043 | <0.016 | 0.051 |
|  | sd | - | 0.0059 | - | 0.019 | - | 0.018 | - | 0.021 |
| 2023 | value | <0.016 | 0.045 | <0.016 | 0.049 | <0.016 | 0.047 | <0.016 | 0.062 |
|  | sd | - | 0.013 | - | 0.010 | - | 0.010 | - | 0.013 |
